# Measuring Three-Dimensional Temperature Distributions in Steel–Concrete Composite Slabs Subjected to Fire Using Distributed Fiber Optic Sensors

**DOI:** 10.3390/s20195518

**Published:** 2020-09-26

**Authors:** Yi Bao, Matthew S. Hoehler, Christopher M. Smith, Matthew Bundy, Genda Chen

**Affiliations:** 1 Department of Civil, Environmental and Ocean Engineering, Stevens Institute of Technology, Hoboken, NJ 07030, USA; yi.bao@stevens.edu; 2 National Fire Research Laboratory, National Institute of Standards and Technology, Gaithersburg, MD 20899, USA; matthew.bundy@nist.gov; 3 Berkshire Hathaway Specialty Insurance, Boston, MA 02111, USA; christopher.smith@bhspecialty.com; 4 Department of Civil, Architectural and Environmental Engineering, Missouri University of Science and Technology, Rolla, MO 65409, USA

**Keywords:** composite structure, distributed fiber optic sensors, fiber optic sensors, fire, high temperature, temperature distribution

## Abstract

Detailed information about temperature distribution can be important to understand structural behavior in fire. This study develops a method to image three-dimensional temperature distributions in steel–concrete composite slabs using distributed fiber optic sensors. The feasibility of the method is explored using six 1.2 m × 0.9 m steel–concrete composite slabs instrumented with distributed sensors and thermocouples subjected to fire for over 3 h. Dense point clouds of temperature in the slabs were measured using the distributed sensors. The results show that the distributed sensors operated at material temperatures up to 960 °C with acceptable accuracy for many structural fire applications. The measured non-uniform temperature distributions indicate a spatially distributed thermal response in steel–concrete composite slabs, which can only be adequately captured using approaches that provide a high density of through-depth data points.

## 1. Introduction

Material temperature distribution can play a significant role in the safety and durability of civil engineering structures. During normal operation, temperature affects the energy efficiency of the building [1] and large temperature gradients can generate or aggravate internal stresses that may cause damage [2]. The damage induced by thermal effects can be particularly critical in large concrete structures, such as dams, because of the significant heat released during the cement hydration process [3]. During an extreme event, such as a building fire, the mechanical properties of construction materials and the load-carrying capacity and stability of structural members (beams, columns, slabs, and joints) are reduced at elevated temperatures [4,5]. Therefore, it is of great importance to assess the temperature distribution of structures at different stages of their life cycle. In this paper, we focus on measurement in fires.

In the literature, methods to assess material temperature distributions during a fire can be grouped into two categories. The first category is based on computer simulations of fire dynamics and heat transfer to predict temperature distributions in structures [6]. Typically, thermodynamic analysis of the fire is performed and followed by heat transfer analysis to the structure to predict the temperature distribution and evolution. A thermo-mechanical analysis is often then performed to analyze the mechanical response of the structure. A number of analytical and numerical tools have been developed to predict gas-phase temperature distributions and evolution histories resulting from fire. Computational fluid dynamics models [7] or stochastic models [8] are often used. Despite these advances, it remains a challenge to accurately predict temperature distributions in structural members using the heat transfer analysis based on gas temperatures and radiative heat flux, in particular for structures with complex geometry such as composite floors [9]. The error in the predicted structural member temperature distribution and evolution over time may result in inaccurate conclusions about the mechanical response of the structure. Additionally, the uncertainty in structural element temperature distribution cannot easily be quantified.

The second category of methods is based on physical measurement of temperature. Infrared cameras can image temperature distributions on the surface of structures [10]. With the recent advent of robotic platforms, such as various unmanned vehicles, infrared cameras have become more attractive for use in hazardous applications. However, infrared cameras only provide measurement of temperature at the visible surface, and cannot measure the internal temperature, which is often important. For instance, the internal temperature of concrete may significantly affect the bond strength between steel and concrete [11]. In current practice, the internal temperature in concrete is frequently measured using thermocouples. Each thermocouple monitors a single point where the thermocouple junction is located. Numerous thermocouples are thus needed to monitor a large structure [12].

Recently, various fiber optic sensors have been developed for structural fire applications. These include fiber Bragg grating (FBG) sensors, long-period fiber grating (LPFG) sensors, interferometer sensors, and distributed fiber optic sensors [13]. FBG sensors and LPFG sensors are manufactured by inscribing gratings (i.e., periodic change of the refractive index) in optical fibers. The gratings change if the fiber is subjected to environmental variations, e.g., changes in fiber temperature. For example, FBG sensors were used to measure gas temperatures up to 300 °C in a scaled (1:20) tunnel [14]. However, conventional FBG sensors are prone to thermal degradation and have a maximum operation temperature of about 350 °C [13]. Regenerated FBG (rFBG) sensors have been developed using a sophisticated fabrication process to improve the thermal stability of the grating and have been applied to reinforced concrete beams in fire testing to measure temperatures higher than 900 °C [15]. Compared with conventional FBG sensors, LPFG sensors have demonstrated better thermal stability, and have been used to study steel frames subjected to elevated temperatures [16]. The LPFG sensors measured temperatures up to 800 °C. Additionally, various interferometer sensors have been developed to measure temperature and strain in high temperature applications [13]. Among them, extrinsic Fabry–Perot interferometer sensors have been used, specifically in structural fire testing [17] to record temperatures up to about 1000 °C. Grating sensors and interferometer sensors provide measurements at discrete points, while distributed sensors provide continuous measurements along the length of an optical fiber without the need for gratings. For example, a single optical fiber was used to obtain continuous measurement along the entire length of a concrete cylinder [18]. In addition to the capability of continuous measurement, a distributed fiber optic sensor utilizes a telecommunication-grade single-mode optical fiber, which serves as both the distributed sensor and the transmission line, making the sensor inexpensive; less than $0.50 USD per meter. Moreover, the distributed sensors eliminate the need for fabrication of gratings or interferometers using special equipment. However, the initial cost of the data acquisition system is often higher than that of the other fiber optic techniques.

If temperatures in structural members during a fire can be measured with sufficiently fine spatial resolution, analyzing the structural response due to thermal loading will become more tractable. Recently, distributed fiber optic sensors have been proven feasible to measure temperature and strain at high temperatures [19,20]. Different distributed sensors have been used to measure temperatures in small-scale steel and reinforced concrete beams subjected to fire. For example, temperature distributions of a small steel beam exposed to combined fire and mechanical loading were measured and enabled an enhanced thermal–mechanical analysis of the steel beam [21]; In addition, temperature distributions in a small reinforced concrete beam exposed to fire were measured and enabled detection of cracks in the concrete [22]. Based on the existing studies, it is rational to hypothesize that distributed fiber optic sensors can be deployed in structures to image three-dimensional temperature distributions and generate digital models of these distributions.

This study has three objectives: (1) develop a method to image three-dimensional temperature distributions in concrete members; (2) develop a method to install distributed sensors following a procedure feasible for building construction practice; and (3) implement and evaluate the methods in six small steel–concrete composite slabs to understand their response under severe fire conditions. The measurements are used to generate three-dimensional information on temperature fields in the concrete and compared with temperatures measured using co-located thermocouples and an infrared camera. The remainder of the paper is organized as follows: Section 2 introduces the experimental program, including the specimens and material properties, instrumentation, test setup, and fire testing protocol; then, Section 3 presents and discusses the experimental results; finally, Section 4 summarizes the new findings from this research.

## 2. Experimental Program

### 2.1. Specimens and Material Properties

**Figure 1:**
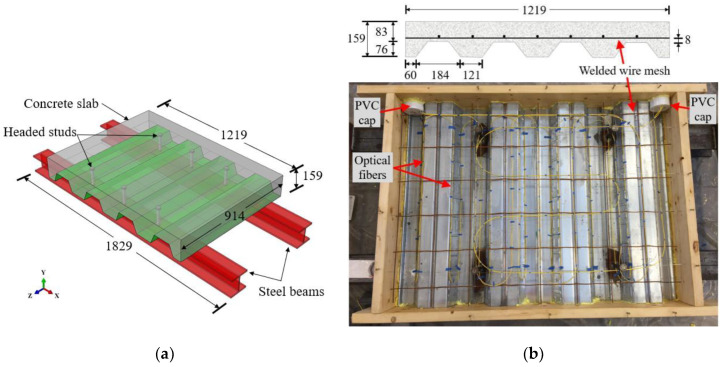
Steel–concrete composite slab specimens (units in mm): (**a**) isometric rendering, and (**b**) cross-sectional and top views. The yellow cables are the optical fibers.

Table 1 summarizes the six composite specimens (CS), designated as CS-1 to CS-6, fabricated to develop the installation scheme of distributed fiber optic sensors (DFOS) in concrete and investigate the response of the distributed sensors to fire-induced temperature increase in concrete. Each DFOS system is compared with a number of thermocouples (TCs) to validate its accuracy. Each specimen consisted of a reinforced concrete slab supported on two W5×19 steel beams, as depicted in Figure 1a. Composite action between the concrete slab and steel beams was achieved using headed steel studs. It is noted that although the number of headed studs varied (four or six), this did not play a significant role in this study.

Each concrete slab was 1219 mm long and 914 mm wide and cast on 0.9 mm thick, trapezoidal metal decking (Vulcraft 3VLI20), which served as stay-in-place formwork. Consequently, the depth of the concrete slab varied from 83 mm to 159 mm, as illustrated in Figure 1b. The concrete slab was reinforced with welded wire mesh (6 × 6, W1.4/W1.4), which had a specified mesh spacing of 150 mm and a wire diameter of 3.4 mm. The headed steel studs had a specified shaft diameter of 19 mm and an effective embedment length of 117 mm. The two W5×19 steel beams were 1829 mm long and placed in parallel with 610 mm spacing.

Each steel–concrete composite specimen was fabricated as follows:

- Each end of the two parallel steel beams was connected by a perpendicular, welded rectangular steel tube so that the beams maintained their position during fabrication.
- A 1219 mm × 914 mm rectangular wood formwork was prepared.
- The trapezoidal metal decking was laid on top of the beams inside the formwork.
- The headed studs were welded through the metal decking to the steel beams (Figure 1).
- The welded wire mesh was supported by plastic chairs 8 mm above the metal decking.
- Optical fibers and thermocouples were deployed as detailed in Section 2.
- Concrete was poured into the formwork. Concrete placement using a hand trowel as well as placement directly from the chute on the concrete truck was used.
- The sides of the formwork were tapped using a rubber mallet to consolidate concrete at the edges; no mechanical vibrators were used.
- The cast specimen was covered under wet burlap and a plastic sheet, demolded after 1 day, and cured at room temperature (22 °C ± 3 °C).

The concrete mix was 0.193: 0.105: 0.315: 1.0: 1.136 by mass for water: Class C fly ash: Type I Portland cement: river sand: expanded slate lightweight aggregate (LWA). With a binder composed of 25% fly ash and 75% Portland cement, this mix corresponded to a water-to-binder ratio of 0.46. The river sand had a diameter up to 4.75 mm. The lightweight aggregate used had low water-retention characteristics and high desorption [23,24,25]. The river sand and lightweight aggregate were prepared to ensure the saturated-surface-dry condition. A polycarboxylate-based high-range water reducer was used to improve flowability of the concrete. The dosage of the water reducer was adjusted to achieve a specified slump of 220 mm to ensure adequate consolidation during casting. Polypropylene monofilament microfibers (FRC MONO-150 ^®^) with a nominal diameter of 38 μm and length between 13 mm and 19 mm were added to the mix at a dosage of 2.37 kg/m^3^ of concrete to reduce temperature-induced spalling [26]. The concrete was mixed at a local batching plant and transported less than 10 min to the test facility for casting.

During concrete casting, ten standard cylinders measuring 102 mm in diameter and 203 mm in height were prepared for concrete material testing. Five cylinders were tested and analyzed to determine the average ± standard deviation of each concrete property. Specifically, the concrete density was 2070 ± 80 kg/m^3^ at 28 days. The compressive strength of concrete was 38 ± 3 MPa at 28 days and 41 ± 3 MPa at 56 days, which were determined in accordance with American Society for Testing and Materials (ASTM) C39/C39M [27]. A relative humidity sensor (Vaisala HM40S RH Probe) was inserted into each concrete slab to measure the internal relative humidity of concrete at a depth of 90 mm. The internal relative humidity measured shortly before the fire test is listed in Table 1.

Table 2 lists the nominal tensile yield strength (*f_y_*), the ultimate strength (*f_u_*), and the modulus of elasticity (*E*) of various types of steel adopted in this study, as specified by the manufacturers.

### 2.2. Instrumentation

**Figure 2:**
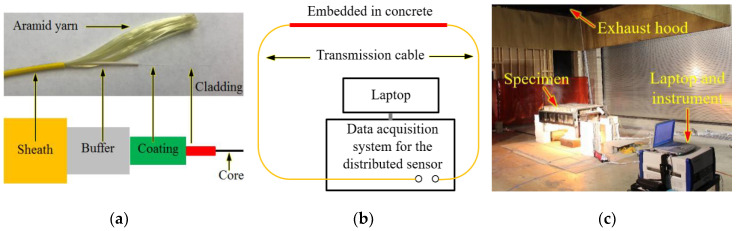
Distributed fiber optic sensor: (**a**) sensor structure, (**b**) schematic of measurement setup, and (**c**) photo of setup. A length of the fiber optic cable was embedded in the concrete slab of the specimen.

The distributed fiber optic sensor (DFOS) used in this study was a simplex fiber optic cable composed of a polymer sheath, an aramid yarn, and an optical fiber, as illustrated in Figure 2a. The optical fiber had a buffer (outer diameter: 880 μm), an outer coating (outer diameter: 242 μm), an inner coating (outer diameter: 190 μm), a glass cladding (outer diameter: 125 μm), and a glass core (diameter: 8.2 μm). The buffer and coatings were made from polymer and used to protect the glass from mechanical impact, and abrasion/environmental exposure, respectively. According to preliminary testing, after the fiber optic cable was embedded in the concrete slab, the optical fiber was free to slide in the sheath with negligible friction so that it was approximately free of axial strain over the relatively short fibers used in this study. Thus, the DFOS can be used to measure temperature changes [32]. As illustrated in Figure 2b, the optical fiber formed a closed loop with a data acquisition system and served as a light transmission cable in addition to sensing. The data acquisition system was set to sample points every 1 cm over a measurement distance of up to 200 m so that the temperature of two points spaced at no less than 2 cm could be distinguished. Figure 2c shows the experimental setup. The instrument was about 10 m away from the specimen during the fire testing. The measurement from the optical fiber was made using a designated instrument, the Neubrescope^®^ (model: NBX-7020), based on Brillouin scattering, as elaborated in a prior study [32]. The instrument measured the temperature-dependent Brillouin frequency shift along the fiber optic cable. In this study, the scanning frequency was swept from 10.82 GHz to 11.67 GHz, which corresponded to an approximate temperature range of 20 °C to 1100 °C [32]. The relationship between the Brillouin frequency shift and temperature was calibrated in prior studies, as elaborated in a prior study [19]. The sensitivity coefficients were determined using a tube furnace with computer-controlled temperature. The calibration relationship is shown in Equation (1):

(1) $$\Delta \nu_{B}=\left(-3.464\times 10^{-7}T+1.110\times 10^{-3}\right)\left(T-22\right)$$

where $\Delta \nu_{B}$ denotes the Brillouin frequency shift (GHz); and *T* denotes temperature (°C). Additional information about the Brillouin scattering technique and fiber calibration are provided in [33].

**Figure 3:**
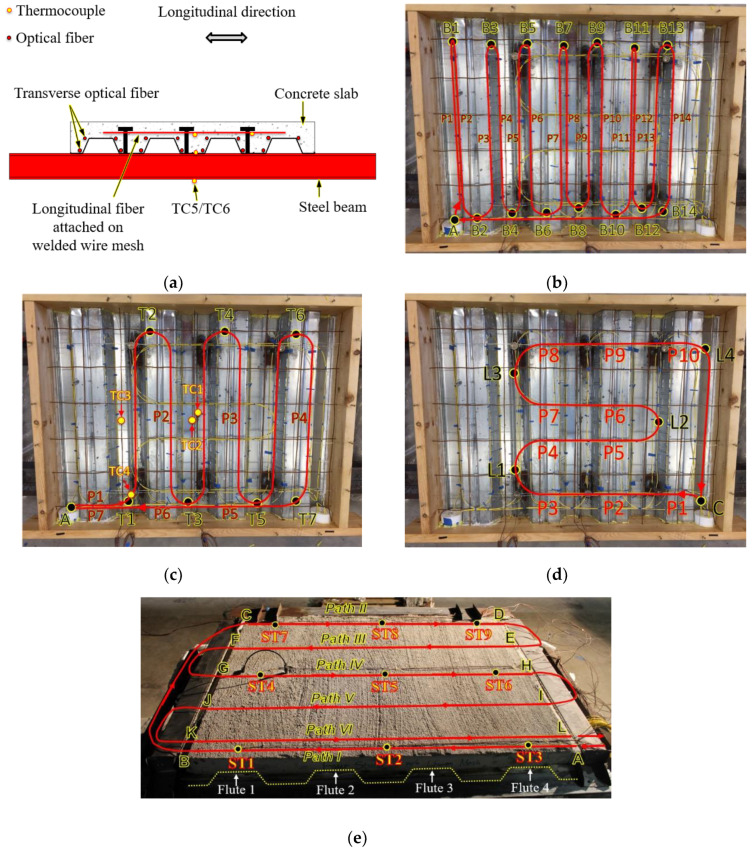
Instrumentation layout: (**a**) elevation view, (**b**) top view of DFOS-1, (**c**) top view of DFOS-2, (**d**) top view of DFOS-3, and (**e**) top view of DFOS-4.

Figure 3 shows the deployment of the sensors. Specimens CS-1 to CS-4 were instrumented with three distributed fiber optic sensors (DFOS-1 to DFOS-3) each. Specimens CS-5 and CS-6 had an additional distributed fiber optic sensor (DFOS-4) on the top surface.

DFOS-1 was laid directly on top of the metal decking along its flutes, which is perpendicular to the steel beams. DFOS-2 was attached on top of the welded wire mesh and also ran parallel to the flutes of the specimen. DFOS-3 was also attached on top of the welded wire mesh but ran perpendicular to the flutes of the specimen. For reference, the curved portions of the distributed sensors were labeled as B1 to B14 for sensor DFOS-1, T1 to T7 for sensor DFOS-2, and L1 to L4 for sensor DFOS-3. The two sensors running transverse to the steel beams (DFOS-1 and DFOS-2) entered the concrete from a polyvinyl chloride (PVC) cap at Point A, and the longitudinal sensor DFOS-3 entered the concrete from a PVC cap at Point C. Each cap measured 100 mm in diameter and was used to store the lengths (500 mm) of each distributed sensor at the two ends during concrete casting and transport of the specimens. The gap between the cup and formwork was small enough to prevent the leaking of concrete. The cup provided mechanical protections and ensured that the distributed sensors were free from the concrete. After the specimens were demolded, the length of each distributed sensor stored in cup was connected to data acquisition system for measurement. Sensor DFOS-4 was attached on the top surface of the concrete after the concrete was cured for one year. A thin groove (depth ≈ 3 mm) was cut into the concrete using a grinding wheel to accommodate the optical fiber and a thin layer (≈1 mm) of cement mortar was used to cover the sensor. The distributed sensor passed across the specimen in six paths perpendicular to the flutes of the slab, denoted by Path I to Path VI, as shown in Figure 3e. All optical fiber turns had a radius larger than 50 mm, which is adequate to avoid any significant light signal attenuation.

Figure 3 also shows thermocouples “TC” embedded in the concrete and thermocouples “ST” attached on the top surface of the concrete slab. In specimens CS-1 to CS-4, each specimen was instrumented with six glass-sheathed, bare bead, K-type thermocouples (24-gauge wire), designated as TC1 to TC6. TC1 was located on the top surface of the metal decking in the center of the specimen. TC2 was on the welded wire mesh in the center of the specimen. TC3 was on the welded wire mesh 305 mm away from the mid-span. TC4 was on a headed stud 300 mm away from the mid-span. TC5 and TC6 were peened into a small drill hole (diameter: 1.5 mm) in the center bottom flanges of the two steel beams. An additional Inconel-shielded thermocouple located 25 mm below the metal decking at the center of the compartment was installed to measure the gas temperature just below the concrete deck. In specimens CS-5 and CS-6, each specimen was instrumented with nine additional thermocouples on the top surface of the concrete slab, designated as ST-1 to ST-9 in Figure 3e. All the thermocouples have a manufacturer-specified temperature standard limit of error of 2.2 °C or 0.75% (whichever is greater) over a measurement range of 0 °C to 1250 °C. The total expanded uncertainties (coverage factor of 2) for the material temperature and gas temperature measurements are ±6.2% and ±14.7% of the reading, respectively. The total expanded uncertainty for the burner heat release rate is less than ±2.4% [34]. The distributed optic fiber sensors have an estimated expanded uncertainty of ±11.2% of the reading. Data from the fuel delivery system and thermocouples were recorded at a rate of 1 Hz.

In addition to the fiber optic sensors and thermocouples, a high-speed mid-wavelength infrared camera (FLIR SC8300HD ^®^) was used to image the surface temperature of select specimens, with emphasis on the top surface of each concrete slab.

### 2.3. Test Setup

**Figure 4:**
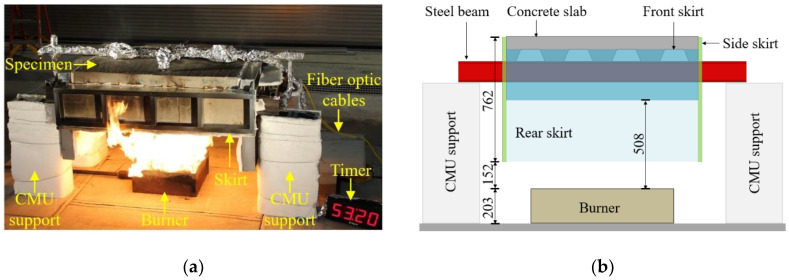
Test setup: (**a**) photograph, and (**b**) illustration (units in mm).

Fire tests were conducted in the National Fire Research Laboratory at the National Institute of Standards and Technology (NIST). The test setup was not intended to represent a particular structure, but rather to investigate the performance of distributed sensors in a typical steel–concrete composite structure. Figure 4 depicts the test setup located under a 6 m × 6 m (plan) exhaust hood (see Figure 2c in Section 2.2). The flame source was a natural gas diffusion burner measuring 530 mm × 530 mm × 200 mm (length × width × height). Natural gas entered the burner near the bottom, filled the burner cavity, and percolated through a gravel layer to distribute the gas. The burners were manually regulated using a needle valve on the gas supply line. A skirt constructed of cold-formed steel framing and cement board lined with thermal ceramic fiber blankets partially enclosed the space above the burner to trap hot gases beneath the specimen. The skirt was open at the bottom, creating the compartment fire depicted in Figure 4a. The heated compartment created by the skirt was approximately 1220 mm × 920 mm × 300 mm (length × width × height). Each beam was simply-supported at a clear-span of 1530 mm on four supports made of stacked concrete masonry units (CMU). The supports were wrapped with 25-mm thick thermal ceramic blankets for thermal protection.

### 2.4. Fire Testing Protocol

**Figure 5:**
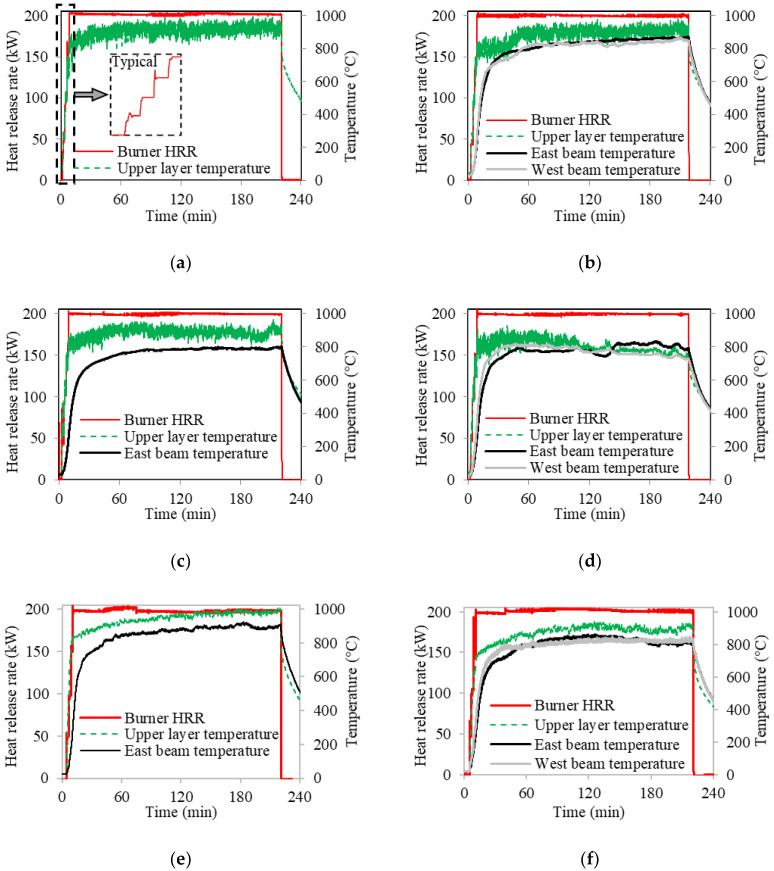
Heat release rate and temperature: (**a**) CS-1, (**b**) CS-2, (**c**) CS-3, (**d**) CS-4, (**e**) CS-5, and (**f**) CS–6.

All specimens were subjected to a similar fire protocol with a peak heat release rate (HRR) of 200 kW. No mechanical load beyond self-weight was applied. The magnitude of fire was controlled through the burner’s calculated heat release rate. Figure 5 shows the burner HRR, compartment upper layer gas temperature, and beam temperatures measured from TC5 and TC6 peened into the bottom flange of the steel beams along the centerline. No thermocouple was deployed on the steel beams of specimen CS-1 and only one beam thermocouple was deployed in CS-3 and CS-5.

The heat release rate was held approximately constant at 50 kW, 100 kW, 150 kW, and 200 kW. Each of the first three levels was maintained for 2 min; the last level (200 kW) was maintained for 210 min, before the fire was extinguished. In total, each specimen was heated for approximately 216 min (3 h 36 min). For specimens CS-1 to CS-3 and CS-6, the upper layer gas temperature was 897 °C ± 38 °C (mean ± standard deviation) when heat release rate was 200 kW, as shown in Figure 5. During the fire test of specimen CS-4, a burner frame weld failed, skewing the flame, and a small fan was used to redirect the fire beginning at 100 min. The draft caused by the fan changed the fire dynamics and resulted in variations of about 100 °C of air and beam temperatures, as shown in Figure 5d. For specimens CS-5 and CS-6, the compartment height was inadvertently reduced by one CMU block (≈200 mm), which resulted in slightly higher upper layer temperature and beam temperature as well as possibly the slightly smaller fluctuation of upper layer temperature, as shown in Figure 5.

## 3. Results and Discussion

### 3.1. Observations

All of the distributed fiber optic sensors survived the installation, concrete casting and curing process without damage. The two methods of concrete placement, careful hand troweling and direct pouring from a concrete truck chute, did not make a difference on the integrity of installed sensors.

**Figure 6:**
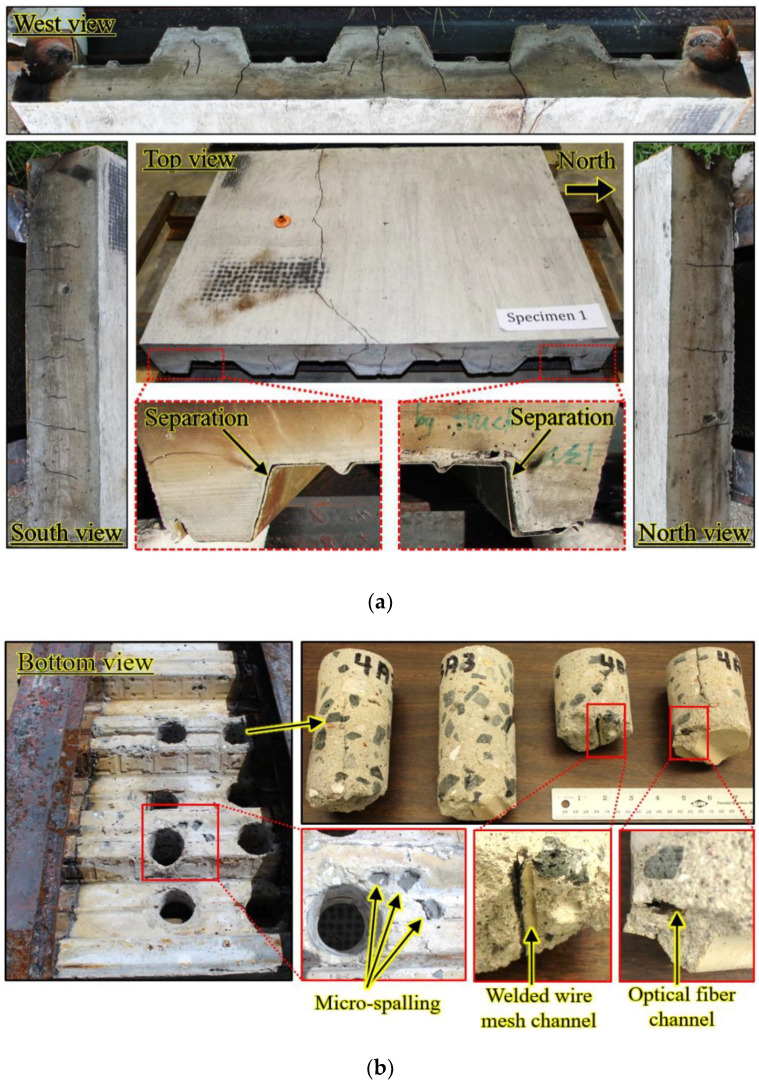
Photograph of CS-1 after fire test: (**a**) before removal of the steel deck, and (**b**) after removal of the metal decking at the bottom of the reinforced concrete slab.

Shortly after a heat release rate of 200 kW was achieved, popping sounds were heard from the specimen; however, no cracking or spalling was observed on visible surfaces. The sounds were attributed to spalling of the concrete at the interface with the metal decking. After approximately 10 min at 200 kW, separation between the metal decking and concrete was observed, as shown in Figure 6a. After the test, when the specimens had cooled, the metal decking was removed for visual inspection. Figure 6b shows the bottom of the concrete slab. Some aggregates were visible and believed to result from “micro-spalling” of concrete adjacent to near-surface aggregate, consistent with the popping sounds. The micro-spalling is attributed to moisture in lightweight aggregates creating internal vapor pressure under fire-induced temperature increase. Diagonal cracks formed near the mid-span of the concrete slab around 30 min after fire ignition. A crack through the specimen width was visible on the top surface at about 40 min. Additional cracks appeared in the specimen until 200 min. Similar phenomena were observed in the other specimens. It is noted that the timing and sequence of the events are specific to the investigated specimens and fire conditions.

After the fire test, several cylinders measuring 76 mm in diameter were cored from the specimens using a diamond core drill, as depicted in Figure 6b. Cylinders were taken in both the thick (thickness: 159 mm) and thin (thickness: 83 mm) sections of the trapezoidal concrete slab. Channels in the concrete created by the removed welded wire mesh and burnt optical fiber sheathing can be observed. While some polypropylene microfibers could still be observed on the top surface of the concrete, no polypropylene fibers could be found near the bottom surface of the tested specimen. This is an indicator of the temperature variation through the depth of the concrete. The melting point of polypropylene fibers is about 165 °C. In spite of the high relative humidity of the concrete (which relates to the moisture content), no widespread spalling occurred, likely due to the use of polypropylene fibers that melted and provided mechanisms for alleviating internal vapor pressure in the concrete slab [35].

### 3.2. DFOS Temperature Measurements

**Figure 7:**
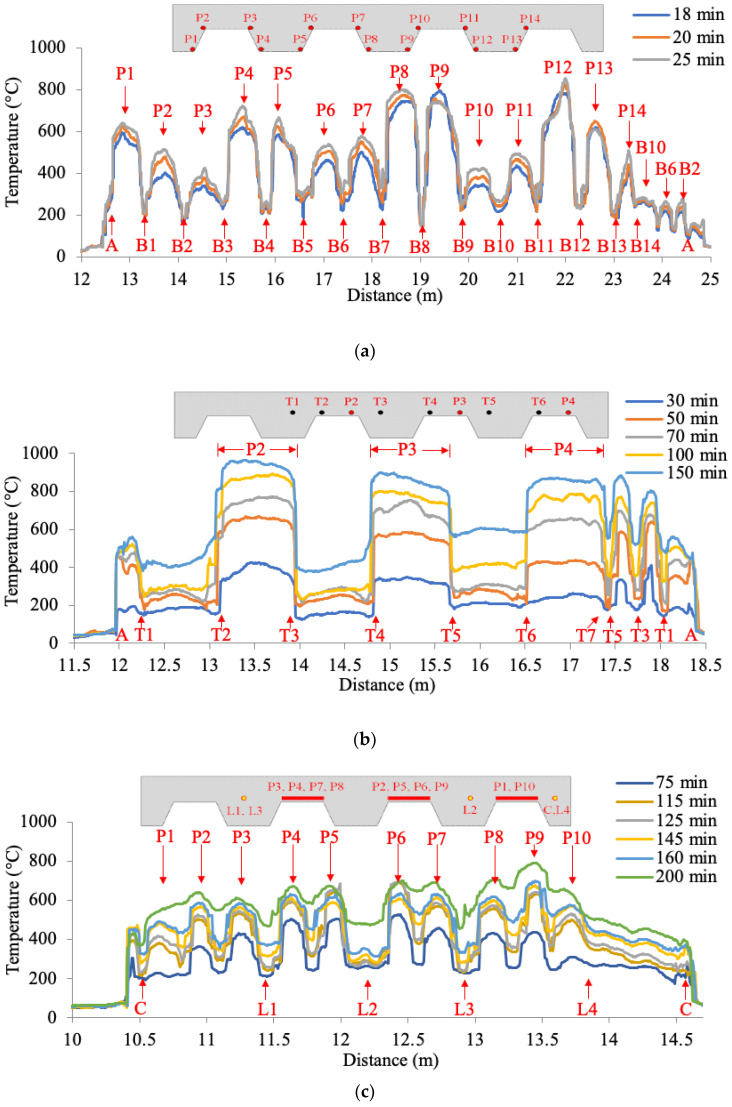
Temperature distributions measured from the distributed sensors in specimen CS-1: (**a**) DFOS-1, (**b**) DFOS-2, and (**c**) DFOS-3. The peaks in slab illustration and test results are marked as “Pn”, where “n” indicates the location of a peak (see Figure 3).

After a constant heat release rate had been achieved, temperature distributions along the length of the distributed sensors DFOS-1, DFOS-2, and DFOS-3 in specimen CS-1 at various time instants are plotted in Figure 7. The times are relative to burner ignition. In each figure, the horizontal axis represents the distance along the distributed sensor, measured from the connection to the data acquisition system. The vertical axis represents the measured temperature, which was obtained from the Brillouin frequency shift measured by the distributed sensor. It can be seen that, as expected, temperature increases with time in all three sensors during the heating phase of the experiment. However, the spatial patterns of temperature distribution observed from the three sensors are quite different. The different patterns are primarily due to the different locations of the sensors deployed in the concrete, as depicted in Figure 3b–d. The locations of the curved portions of the distributed sensors are marked in Figure 7 for reference.

In Figure 7a, the first 14 peaks are marked as P1 to P14 along the centerline of the specimen. DFOS-1 measured the temperature in the bottom of the concrete slab just above the steel deck. The fact that the peaks occurred along the centerline suggests that: (1) the gas temperature and radiative heat flux were lower near the edges of the test setup; (2) there was more heat loss from the specimen to the surrounding environment at its edges; and (3) the steel beams near the edges provided heat sink for the concrete slab. The temperature variation transversely across the specimen is significant: over 600 °C variation for fiber section B8 above a steel beam. This spatial variation has been neglected in thermo-mechanical analysis where it is typically assumed that gas temperature and heat flux below the slab is uniform. P8 and P9 above the burner exhibited the highest temperatures. Overall, the temperatures at P1, P4, P5, P8, P9, P12, and P13 are higher (on the order of 200 °C to 400 °C) than the temperatures at P2, P3, P6, P7, P10, P11, and P14. This comparison suggests that the lower flanges of the concrete slab were subjected to more intense thermal conditions than the higher flanges because they were closer to the burner. The peaks after P14 are marked by the locations of the curved portions of the distributed sensor. For instance, the peak B10, which is the peak after P14 in Figure 7a, corresponds to the portion of the distributed sensor near B10, as seen in Figure 3b. Unlike the previous portion of DFOS-1, the optical fiber ran from B14 to A in a straight line and at a constant height in the concrete slab. The peaks B10, B6, and B2 are therefore associated with the change of the thickness of the concrete slab. Because of the varying thickness of the concrete slab, the distributed sensor at the locations B10, B6, and B2, which are above the upper flanges of the decking, was closer to the metal decking (and thus the hot compartment) than that at the locations B12, B8, and B4.

In Figure 7b, the peaks marked by P2 to P4 represent the locations of sensor DFOS-2 parallel to the flute above the thin portions of the deck profile, as depicted in Figure 3c. DFOS-2 measured temperature along the welded wire mesh (the optical fiber was taped to the mesh to maintain its position prior to concrete casting), which was about 8 mm away from the high flute of the metal decking, as illustrated in Figure 1b. Because the welded wire mesh was at a roughly constant height in the concrete slab, the temperature is higher in the optical fiber section above the upper flange of the metal decking (8 mm concrete cover) than that in the section above the lower flange of the metal decking (84 mm concrete cover). In each of the peaks P2 to P4, the temperature distribution is approximately constant and the temperature at the centerline is not significantly higher than that at the specimen sides, which is different from the observations in Figure 7a. This difference is attributed to three mechanisms. First, the concrete cover serves as a thermal “buffer” that smooths the temperature gradient. Second, the high thermal conductivity of the steel wire mesh (to which the fiber was taped) further reduced any temperature gradient along the fiber. Third, delamination of metal decking decelerated the heat transfer from the metal decking to the concrete slab, reducing the temperature gradient in the concrete slab. Other peaks besides P2 to P4 were due to the geometry of the metal decking. Although a simultaneous comparison of the temperatures in DFOS-1 and DFOS-2 was not possible due to the required data acquisition time for each fiber loop (roughly 5 min), comparing the temperatures between B6 and B7 in DFOS-1 at 25 min and the temperatures between T2 and T3 in DFOS-2 at 30 min (which are roughly comparable in time and horizontal position in the concrete slab), it can be seen that the average temperatures are slightly higher in DFOS-1 (374 °C) than in DFOS-2 (369 °C), which is expected based on the concrete cover for each fiber sensor. At 150 min, a temperature up to 960 °C was measured at P2.

The first 10 peaks in Figure 7c are marked by P1 to P10, which represent the lengths of sensor DFOS-3 in the vicinity of the metal decking, as depicted in Figure 3d. DFOS-3 measured the temperature along the welded wire mesh perpendicular to the direction of the decking flutes. Along the length of DFOS-3, the distance between the fiber optic sensor and the metal decking varied. The temperature is higher in the portions of the sensor that are close to the metal decking than in the portions that are far from the decking. As the fire testing time increases from 160 min to 200 min, the peaks become less prominent. This trend indicates that the temperature gradient in the concrete slab decreases over time as the temperature of the entire concrete volume increases.

**Figure 8:**
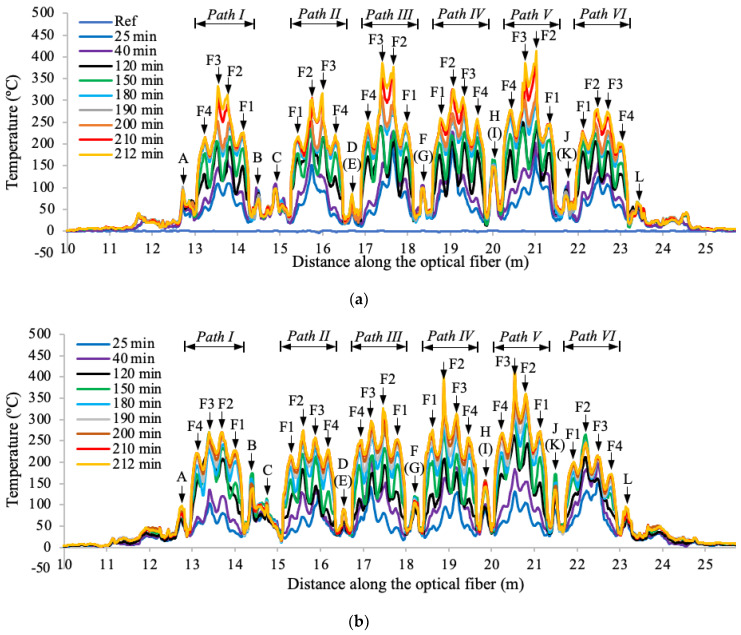
Surface temperature distributions measured from DFOS-4 in: (**a**) CS-5, and (**b**) CS-6. “F1” to “F4” stand for Flute 1 to Flute 4 shown in Figure 3e.

Figure 8 shows the surface temperature distributions measured from DFOS-4 sensors in specimens CS-5 and CS-6, respectively. In each specimen, the distributed sensor measured the temperature distributions along the six paths on the top surface shown in Figure 3e. Within the range of each path, four peaks are identified corresponding to the four upper flutes of the concrete deck (F1 to F4), indicating a higher surface temperature at the thin concrete sections; by comparing the temperatures corresponding to each flute in the same path, it is seen that the Flute 2 and Flute 3 have higher temperatures than Flute 1 and Flute 4, because the middle of the slab directly above the burner experienced higher temperature than the two ends of the slab, consistent with the observations from Figure 7.

### 3.3. Discussion

**Figure 9:**
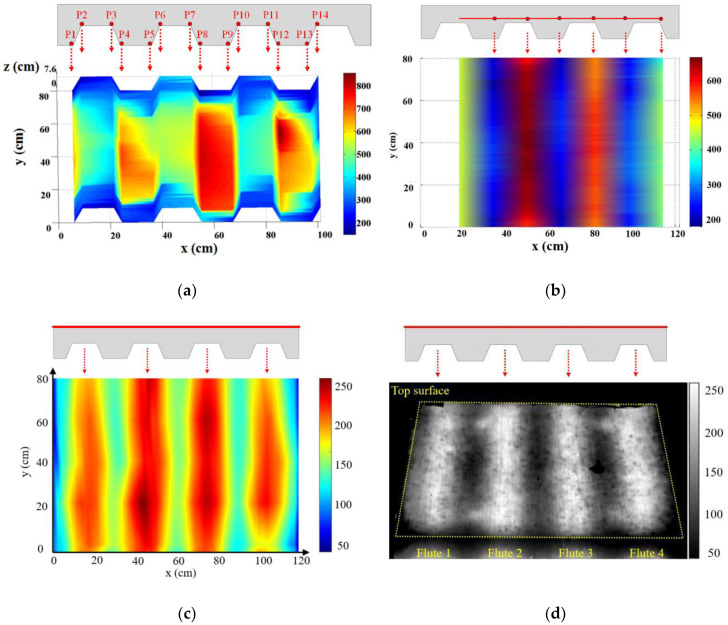
Visualization of temperature measured from: (**a**) DFOS-1 in CS-1 at 25 min, (**b**) DFOS-2 in CS-1 at 50 min, (**c**) DFOS-4 in CS-5 at 150 min, and (**d**) infrared camera for CS-3 at 180 min. The legends show temperature in °C. The red lines and dots represent various distributed sensors, respectively.

As demonstrated in Section 3.2, the distributed fiber optic sensors provide a wealth of information regarding the local spatial temperature variation in the specimen. To help understand this data, the temperatures measured along the distributed sensor can be plotted for visualization using the position of a distributed sensor in a composite specimen. The results are presented in Figure 9a for sensor DFOS-1 in specimen CS-1 at 25 min, Figure 9b for sensor DFOS-2 in specimen CS-1 at 50 min, Figure 9c for sensor DFOS-4 in specimen CS-5 at 150 min, and Figure 9d for the infrared camera image in specimen CS-3 at 180 min. The spatial distribution of temperatures in concrete slabs is useful to understand and model the specimen behavior. All of the visualizations in Figure 9 clearly show the periodic temperature variation in the concrete resulting from the varying thickness of the trapezoidal concrete slab. As noted in Section 3.2, but more clearly visible in Figure 9a, the temperatures at the outside edges of the slab near the bottom were significantly lower than between the steel beams at the center of the slab. This effect was less pronounced as the measurement positions moved up in the slab, as seen by comparing Figure 9b to Figure 9c. These through-depth and in-plane temperature variations would only be captured with a very dense array of the conventional thermocouples.

**Figure 10:**
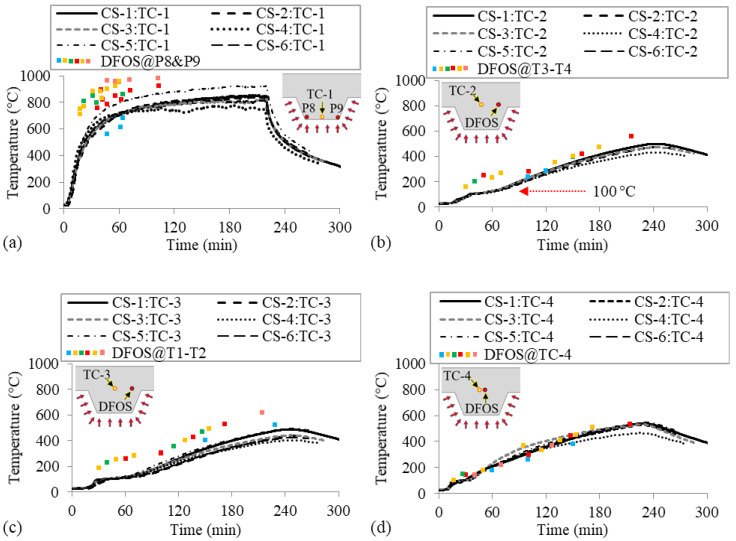
Temperature histories in the tested specimens using: (**a**) TC-1, (**b**) TC-2, (**c**) TC-3, and (**d**) TC-4. The illustrations depict the positions of the thermocouple and distributed sensors.

Figure 10 shows the temperature histories measured by the thermocouples and distributed sensors in all six specimens. The results obtained from the distributed sensors of different specimens are represented by the squares in different colors. Since the distributed sensors did not precisely pass by the thermocouples, the thermocouple results measured by TC-1 to TC-4 are compared with the temperatures measured from the nearest point along the distributed sensors. The horizontal distances between the reported points in the distributed sensors and the thermocouples TC-1 to TC-3 were about 60 mm and their vertical position in the concrete slab was similar (±2 mm). Their positions in the concrete slab are illustrated in the insets in Figure 10. The horizontal distance between TC-4 and the reported point in distributed sensor DFOS-2 was about 10 mm.

Figure 10a shows the temperature histories measured from thermocouple TC-1 and mean temperatures over 30 s increments measured by the distributed sensors at P8 and P9, as shown in Figure 3b, for all six specimens. Among the four thermocouples in each specimen, TC-1 measured the highest temperatures because it was located closest to the metal decking (smallest concrete cover) and directly above the burner. The temperatures from TC-1 in specimen CS-4 decreased slightly after 100 min because of the fan used to redirect the flame during the fire test, as discussed in Section 2.4, and the temperatures in specimen CS-5 were slightly higher than the other specimens due to the inadvertent reduction in the compartment height for that test, as reflected in gas temperature histories in Figure 5. The DFOS measurements stopped by 120 min because the optical fibers at the interface with the steel deck were destroyed by this time; likely due to the micro-spalling of the concrete at this interface fracturing the fiber. At this location, prior to failure, the temperatures measured by the distributed sensors are higher than those measured by the thermocouples. This behavior is consistent with the different positions of the distributed sensors and TC-1, as illustrated in the inset in Figure 10a. The distributed sensors at P8 and P9 were installed at the corner of the deck flute, and thus were subjected to heating from two sides, whereas TC-1 was only subjected to heating from the bottom. It is also because the concrete cover at P8 and P9 varied slightly from that of TC1, which for small cover depths could result in significant temperature difference.

Figure 10b compares the temperature histories measured from TC-2 in all specimens and the temperatures measured from the distributed sensors at the center of points T3 and T4 for the specimens, as shown in Figure 3c. Due to the relatively low thermal conductivity of concrete, the rates of temperature increase at TC-2, TC-3 and TC-4 were lower than that at TC-1. In Figure 10b, the plateau at about 100 °C in the temperature histories measured from TC-2 is caused by water evaporation, which is an endothermic reaction that temporally halts the increase in temperature in the concrete. The temperatures measured by TC-1 in Figure 10a did not exhibit an obvious plateau, because the concrete cover was nearly zero and the moisture evaporation front quickly moved above the thermocouple. The distributed fiber optic sensor results shown in Figure 10b do not exhibit a temperature plateau at 100 °C, as would be expected from the thermocouple measurements. In fact, they indicate a plateau at about 250 °C around 60 min. The reason for this behavior is unknown but may be due to the temporal and spatial averaging used to process the fiber optic data smoothing sharp gradients. Further study of this is required. Additionally, the temperatures measured by the distributed sensors in Figure 10b rise more rapidly than those measured by the related thermocouples for the first 90 min, which is consistent with variations in position of the sensors, as shown in the figure inset.

Figure 10c compares the temperature histories measured from TC-3 and temperature results measured from the distributed sensors at the center of points T1 and T2, as shown in Figure 3c. These locations follow similar trends to those observed above for Figure 10b.

Figure 10d compares the temperature histories measured from TC-4 and the distributed sensors passing by TC-4 at T1 in the specimen. The temperatures and the rate of increase measured from TC-4 and the distributed sensors were similar because the DFOS and thermocouple were co-located.

**Figure 11:**
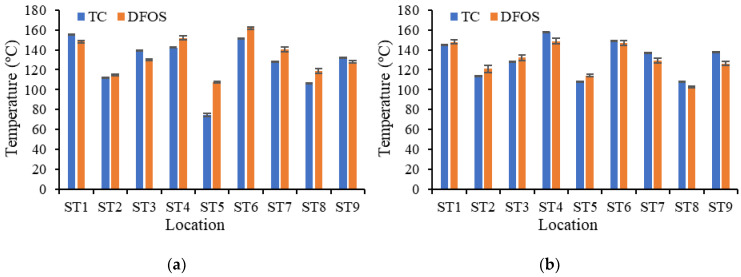
Comparison of temperature measurements from thermocouples (TC) and distributed fiber optic sensors (DFOS) deployed on the top surface of the concrete at 120 min: (**a**) CS-5, and (**b**) CS-6. The error bars depict the standard deviation of the temperature measurement results.

For further comparison between the thermocouples and distributed sensors, the surface temperature measurements from CS-5 and CS-6 at 120 min are plotted in Figure 11. These measurements were made at identical locations (±2 mm) and thus provide a direct comparison of the measurement variability. The locations of ST1 to ST9 are depicted in Figure 3e, and the temperature distributions measured from the distributed sensors are plotted in Figure 9c. The thermocouple results are the mean value and standard deviation of the 60 measurements between 119.5 min and 120.5 min. The distributed sensor results are the mean value and standard deviation of three measurements between 119.5 min and 120.5 min. As discussed in Section 2, the thermocouples had higher sampling rates than the distributed fiber sensors. The significant discrepancy of 33 ºC (30.6% of reading value), which occurs at ST5 of specimen CS-5 in Figure 11a, was due to detachment of thermocouple ST5 from the concrete shortly after fire ignition. Neglecting location ST-5 of CS-5, the median discrepancy of co-located measurements was 6.6% of the reading value. The median coefficient of variation (CoV) for the thermocouples and DFOS were 0.2% and 1.5%, respectively. The maximum discrepancy was 11.9% of the reading value, which occurred at ST-8 for CS-5.

## 4. Conclusions

In this study, temperature distributions were measured using distributed fiber optic sensors installed in steel–concrete composite slab specimens exposed to fire. Compared with traditional point sensors, e.g., thermocouples, this approach provided significantly higher spatial resolution of temperatures.

This limited set of data suggests that the investigated polymer-sheathed optical fibers survived, during the concrete casting process in typical building construction. The temperatures measured using the distributed sensors were in reasonable agreement with the results from thermocouples deployed close to the distributed fiber optic sensors. The measured temperature discrepancies between the fiber optic sensors and the thermocouples was attributed to position differences in the specimens. Further study is recommended to verify this. Material temperatures up to 960 °C were measured at the interface between the concrete and the metal deck.

The measured temperatures from a distributed fiber optic sensor indicate highly non-uniform patterns of temperature distribution in each composite slab specimen, which are often neglected in engineering design and analysis. Deploying the distributed fiber optic sensors in large-scale structural fire tests has the potential to improve our understanding of the performance of infrastructure in fires and, thus, fire safety.

## Figures and Tables

**Table 1:** Details of the composite specimens.

Designation	Age at Testing (days)	Internal Relative Humidity before Test	Number of Studs	Distributed Sensors	Thermocouples
CS-1	33	94.3%	6	DFOS-1 to DFOS-3	TC1 to TC6
CS-2	34	93.5%	6	DFOS-1 to DFOS-3	TC1 to TC6
CS-3	35	95.0%	4	DFOS-1 to DFOS-3	TC1 to TC6
CS-4	36	95.2%	4	DFOS-1 to DFOS-3	TC1 to TC6
CS-5	350	77.7%	4	DFOS-1 to DFOS-4	TC1 to TC6; ST1 * to ST9
CS-6	351	78.6%	6	DFOS-1 to DFOS-4	TC1 to TC6; ST1 to ST9

* “ST” stands for “surface thermocouples”.

**Table 2:** Nominal mechanical properties of steel used in composite specimens.

Steel Used in the Specimens	ASTM Material Standards	Tensile Yield Strength (MPa)	Ultimate Strength (MPa)	Modulus of Elasticity (GPa)	Testing Standard
Beams	A992 (structural steel)	345	450	200	[28]
Headed studs	A108 (cold drawn)	414	496	205	[29]
Welded wire mesh	A185 Grade 65	448	517	200	[30]
Galvanized metal decking	A611 Grade D (cold rolled)	276	359	203	[31]
